# Supplementary material for: The genome sequence of the wood gnat, Sylvicola cinctus (Fabricius, 1787)
Source: Wellcome Open Res. 2025 Feb 28;10:114. [Version 1] doi: 10.12688/wellcomeopenres.23762.1 (PMC12515361; doi:10.12688/wellcomeopenres.23762.1)
Supplement: Peer review history

# Reviewer report: Reviewer response for version 1

**DOI:** 10.21956/wellcomeopenres.26210.r134252

**Published:** 2025-10-11

**Authors:** Panagiotis Ioannidis

**Affiliations:** 1 Foundation for Research & Technology - Hellas, Crete, Greece

This paper describes the sequencing and assembly of the genome of the gnat *Sylvicola cinctus*. All of methodology is the standard one used in other DToL projects. Generally, the genome is of high quality and suitable to be used for downstream analyses.

 However, one thing I noticed is the relatively low BUSCO scores; 93% is kind of low compared to other assemblies I've recently reviewed. Maybe the authors were a bit too aggressive with decontamination or with purging and collapsing contigs? A nice test would be running BUSCO  on the contigs (i.e. before scaffolding) and see if they get a better score...

Are sufficient details of methods and materials provided to allow replication by others?

Yes

Is the rationale for creating the dataset(s) clearly described?

Yes

Are the datasets clearly presented in a useable and accessible format?

Yes

Are the protocols appropriate and is the work technically sound?

Yes

Reviewer Expertise:

insect genomics; bioinformatics

I confirm that I have read this submission and believe that I have an appropriate level of expertise to confirm that it is of an acceptable scientific standard.

# Reviewer report: Reviewer response for version 1

**DOI:** 10.21956/wellcomeopenres.26210.r119926

**Published:** 2025-03-18

**Authors:** Anthony Bayega

**Affiliations:** 1 McGill University, Montreal, Canada

Sivell et al. provide an overview description of the genome of *Sylvicola cinctus *which they sequenced and assembled. I commend them for their efforts. My comments follow below:

 1. In Figure 3, the label on the Y-axis which reads “ERR1…” could be changed to something more informative to help the reader make sense of this figure.

 Overall, the authors provide a high-quality genome and also assign 99.95% of it to chromosomes. Although much work remains to order the scaffolds, sort out the orientation of contigs in the centromere, and complete the gaps and also structurally and functionally annotate the genome, the current work will indeed be valuable to the whole community. I therefore recommend the indexing of this genome so that this resource becomes widely accessible to the scientific community.

Are sufficient details of methods and materials provided to allow replication by others?

Yes

Is the rationale for creating the dataset(s) clearly described?

Yes

Are the datasets clearly presented in a useable and accessible format?

Yes

Are the protocols appropriate and is the work technically sound?

Yes

Reviewer Expertise:

Genomics and Human genetics

I confirm that I have read this submission and believe that I have an appropriate level of expertise to confirm that it is of an acceptable scientific standard.
